# Supplementary material for: Soil fungal communities differ between shaded and sun-intensive coffee plantations in El Salvador
Source: PLoS One. 2020 Apr 24;15(4):e0231875. doi: 10.1371/journal.pone.0231875 (PMC7182172; doi:10.1371/journal.pone.0231875)
Supplement: S2 Fig — (DOCX) [file pone.0231875.s002.docx]

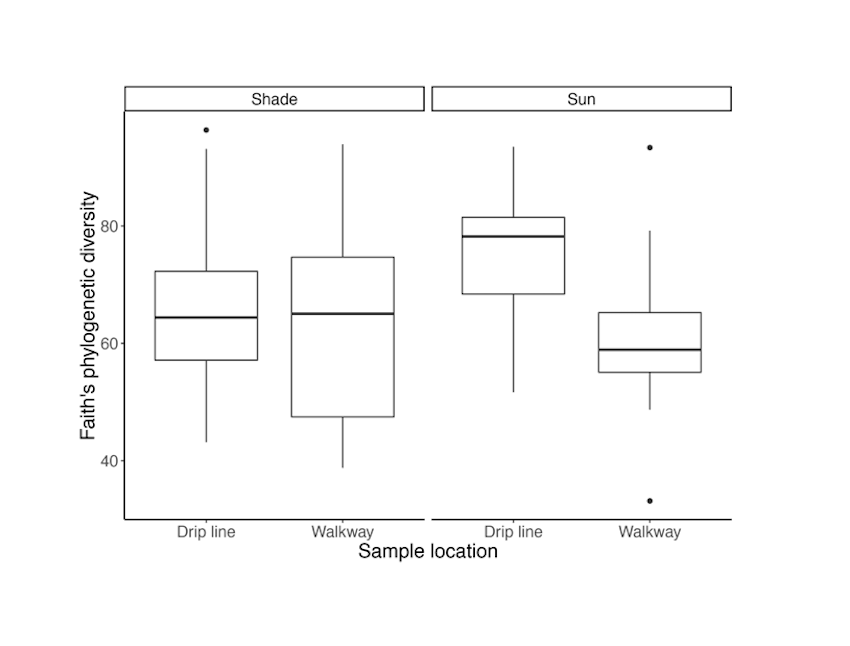


**Figure S2.** Boxplots of overall fungal phylogenetic diversity by system and sampling depth in sun and shade coffee soil.
